# Natural Variation for Responsiveness to flg22, flgII-28, and csp22 and *Pseudomonas syringae* pv. *tomato* in Heirloom Tomatoes

**DOI:** 10.1371/journal.pone.0106119

**Published:** 2014-09-02

**Authors:** Selvakumar Veluchamy, Sarah R. Hind, Diane M. Dunham, Gregory B. Martin, Dilip R. Panthee

**Affiliations:** 1 Department of Horticultural Science, North Carolina State University, Mountain Horticultural Crops Research and Extension Center, Mills River, North Carolina, United States of America; 2 Boyce Thompson Institute for Plant Research, Ithaca, New York, United States of America; 3 Department of Plant Pathology and Plant-Microbe Biology, Cornell University, Ithaca, New York, United States of America; University of the West of England, United Kingdom

## Abstract

Tomato (*Solanum lycopersicum* L.) is susceptible to many diseases including bacterial speck caused by *Pseudomonas syringae* pv. *tomato*. Bacterial speck disease is a serious problem worldwide in tomato production areas where moist conditions and cool temperatures occur. To enhance breeding of speck resistant fresh-market tomato cultivars we identified a race 0 field isolate, NC-C3, of *P. s.* pv. *tomato* in North Carolina and used it to screen a collection of heirloom tomato lines for speck resistance in the field. We observed statistically significant variation among the heirloom tomatoes for their response to *P. s.* pv. *tomato* NC-C3 with two lines showing resistance approaching a cultivar that expresses the *Pto* resistance gene, although none of the heirloom lines have *Pto*. Using an assay that measures microbe-associated molecular pattern (MAMP)-induced production of reactive oxygen species (ROS), we investigated whether the heirloom lines showed differential responsiveness to three bacterial-derived peptide MAMPs: flg22 and flgII-28 (from flagellin) and csp22 (from cold shock protein). Significant differences were observed for MAMP responsiveness among the lines, although these differences did not correlate strongly with resistance or susceptibility to bacterial speck disease. The identification of natural variation for MAMP responsiveness opens up the possibility of using a genetic approach to identify the underlying loci and to facilitate breeding of cultivars with enhanced disease resistance. Towards this goal, we discovered that responsiveness to csp22 segregates as a single locus in an F_2_ population of tomato.

## Introduction

Plants have numerous responses to pathogen attack, including the production of reactive oxygen species (ROS), signaling activated by salicylic acid and jasmonic acid, increased expression of immunity-related genes and, in some cases, development of a hypersensitive response, a form of programmed cell death at the site of attempted infection [1]–[5]. During the initial stages of the interaction plants use pattern recognition receptors (PRRs) to detect pathogen- or microbe-associated molecular patterns (PAMPs or MAMPs), which are typically conserved among microbes [6], [7]. A second plant surveillance system involves intracellular resistance (R) proteins that have evolved to either directly or indirectly recognize pathogen virulence proteins (effectors) that are delivered into the host cell [8], [9]. The R proteins, which activate effector-triggered immunity, are typically present in only certain accessions of a crop species. In contrast, PRRs are often present in all genotypes of a certain species [6], [10]. Bacterial MAMPs include those derived from flagellin (flg22 and flgII-28), cold-shock protein (Csp22), elongation factor Tu (EF-Tu), and lipopolysaccharides (LPS) [11]–[15].

In plants, PRRs are typically receptor-like proteins or receptor-like kinases [3], [7]. Upon recognition of a MAMP the PRR triggers a myriad of defense responses including the production of ROS, changes in gene expression, cell wall reinforcement by callose deposition, a calcium burst and activation of MAPKs (mitogen-activated protein kinases) [3], [16]–[18]. These responses collectively arrest pathogen multiplication. Perception of flg22 has been studied in detail and is known to occur via the PRR FLS2 [19]. Similar to flg22, flgII-28 elicits defense-related responses in tomato such as an increase in the production of the stress hormone ethylene and a rapid production of ROS [11]. flgII-28 appears to be specifically recognized by Solanaceae species [11]. More recently, it has been predicted that there is an additional and yet-to-be identified receptor, FLS3 (Flagellin sensing 3), which is involved in the perception of flgII-28 in the Solanaceae family [20]. It has been reported that suspension cells of *Solanum peruvianum* respond to Csp22 in an alkalinization assay, although there have been no further reports on this system [13].

Tomato (*Solanum lycopersicum* L.) is an important vegetable crop in the world with high nutritional value and versatile food use. Presently, about 100 million tons of fresh market tomatoes are produced on 3.7 million hectares worldwide. The United States is the second largest tomato producing country after China (http://faostat.fao.org/). Tomato cultivars are classified as fresh market, processing, or heirloom (also called vintage) lines. Processing tomatoes are cultivated as a field crop, whereas fresh market and heirloom tomatoes are grown as either outdoor or indoor crops. Heirloom tomatoes are defined as open pollinated cultivars that originated before 1951 [21]. These varieties are highly prized among horticulturalists and home gardeners, and have proven to be a rich source of natural variation for fruit shape and size among other traits [22]–[24]. However, few studies have been conducted to assess the response of heirloom tomatoes to pathogens, although it has been noted that many are susceptible to various diseases [22], [23].

*Pseudomonas syringae* is a hemibiotrophic pathogen classified into more than 50 pathovars based primarily on host range [25], [26]. In tomato, *Pseudomonas syringae* pv. *tomato* (*Pst*) is the causal agent of bacterial speck disease, which is characterized by necrotic spots often surrounded by chlorotic halos caused by the bacterial toxin coronatine [26], [27]. Some tomato cultivars carry the *Pto* gene, which confers resistance to bacterial speck and was originally derived from a wild relative of tomato, *Solanum pimpinellifolium* [28], [29]. The product of *Pto* is a protein kinase that acts in concert with the NB-LRR protein Prf to recognize either of the *Pst* effector proteins AvrPto or AvrPtoB in order to activate ETI [27], [28], [30]. Among *Pst* strains, two races have been identified that differ in their ability to successfully mount an infection in *Pto*-expressing tomato [27], [31]. Race 0 strains are unable to cause disease on *Pto*-expressing tomatoes because they express either or both AvrPto and AvrPtoB (also known as HopAB2). These effectors are translocated into the plant cell where they are recognized by the Pto kinase to activate a strong immune response [32]. Race 1 strains do not express effector proteins recognized by Pto and can cause disease on *Pto*-expressing tomatoes [31], [33]. The *Pto* locus has been incorporated into many processing tomato cultivars and has provided durable control of bacterial speck disease under field conditions [27]. While present in many cultivars of processing tomatoes, *Pto* has been introgressed into relatively few fresh market tomatoes and none of the cultivars derived from the North Carolina tomato breeding program carry the *Pto* gene.

The identification and use of genetic resistance to diseases is desirable as it can provide effective control with reduced pesticide usage. Before the discovery of *Pto*, bacterial speck was mostly controlled by copper-based bactericides and various integrated management strategies [27], [34], [35]. The fact that copper resistant *Pst* strains have arisen [36] as well as an increased public concern about the detrimental environmental effects of pesticide residues and their ineffectiveness prompted us to search for alternative approaches to identify the genetic resistance to bacterial speck.

Here we describe the characterization of a strain of *Pst* from an important tomato-growing region of North Carolina and its use for assessing whether natural variation for speck resistance exists in a collection of heirloom tomato lines. We also examine whether these lines showed variation for their responsiveness to three bacterial MAMPs: flg22, flgII-28 and csp22. Substantial natural variation was observed for these immunity-associated traits thus laying the foundation for using heirloom germplasm to facilitate breeding of speck resistance cultivars as well as for map-based cloning of the genes conferring differential responsiveness to bacterial MAMPs.

## Results

### Collection of *Pseudomonas syringae* pv. *tomato* isolates from western North Carolina

**Figure 1:**
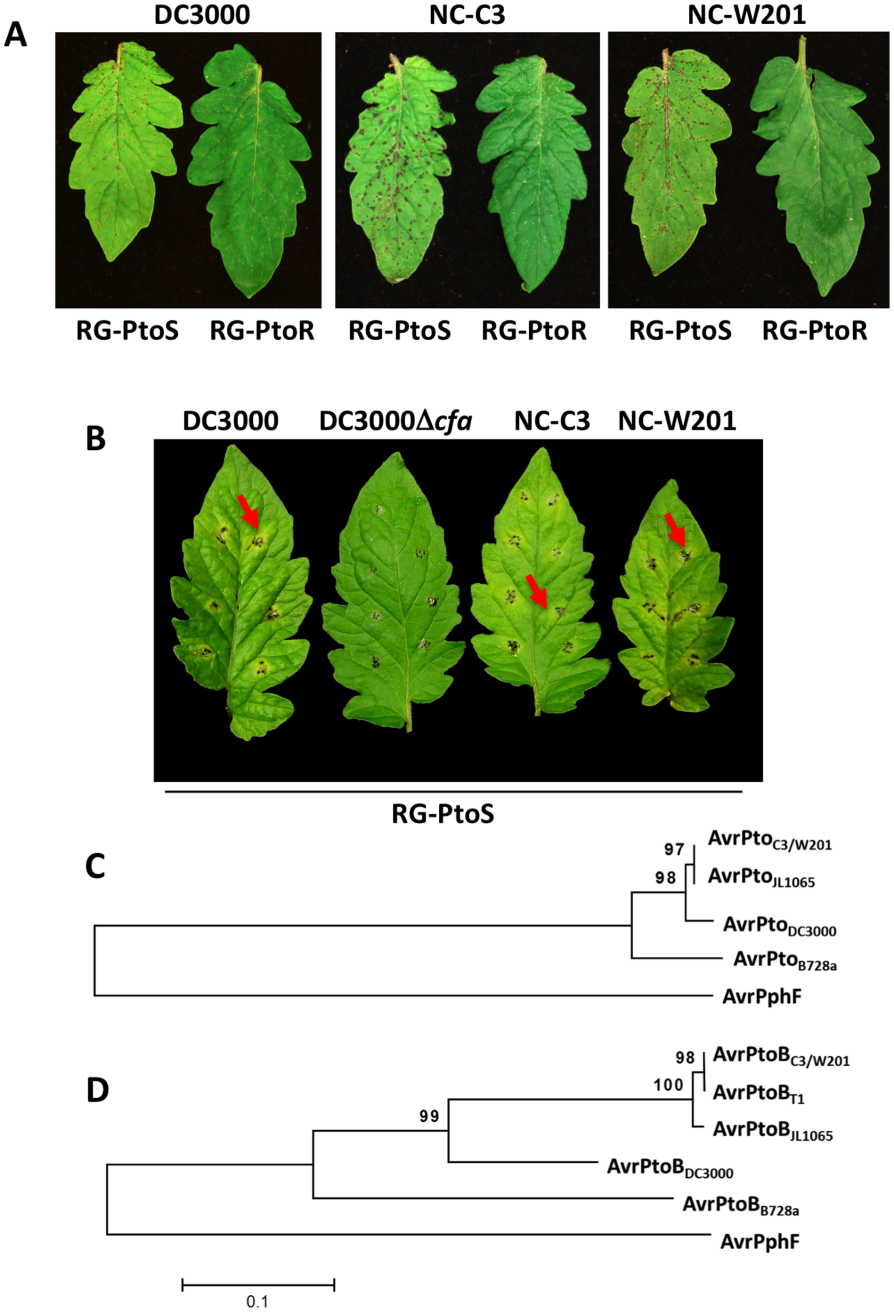
Pathogenicity of North Carolina isolates on tomato, coronatine production and characterization of their AvrPto and AvrPtoB proteins. **A**) The North Carolina isolates NC-C3 and NC-W201 caused bacterial speck disease on leaves of Rio Grande-PtoS (*pto/pto*) plants but not on RG-PtoR (*Pto/Pto*) indicating they are *Pst* race 0 strains like DC3000. Photos were taken 4 days after vacuum-infiltration of 1×10^4^ CFU/mL of the strains indicated. **B**) Both NC-C3 and NC-W201 caused necrotic specks surrounded by diffuse yellow chlorosis indicative of coronatine production similar to DC3000 on leaves of RG-PtoS. The strains were cultured on mannitol-glutamate (MG) media to induce coronatine production prior to inoculation. DC3000Δ*cfa* (CUCPB5502) is a coronatine-deficient mutant used as a control. Photos were taken 5 days after inoculation. **C**) Phylogenetic tree of the AvrPto proteins from the North Carolina strains and other *Pst* strains. **D**) Phylogenetic tree of the AvrPtoB proteins from the North Carolina strains and other *Pst* strains. The NC-C3 and NC-W201 sequences were identical and are therefore grouped together. Parameters for the analysis were neighbor-joining, 10,000 bootstrap replications and p-distance with AvrPphF used as an out-group. The bar indicates the average number of amino acid substitutions/distance. See Figure S1 for an alignment of the AvrPto and AvrPtoB amino acid sequences.

In order to assess the response in the field of heirloom tomato lines to *P. s.* pv. *tomato* (*Pst*) we first sought to collect and characterize an isolate that occurs naturally in North Carolina. Samples were collected from tomato plants having characteristic bacterial speck disease from two western North Carolina counties. After preliminary analysis of the isolates, we focused on two for further testing, NC-C3 and NC-W201 (see Methods and **Table S1**). We observed fluorescence under a UV light when these isolates were grown on KB media, suggesting they are *Pst*; as expected, the well-characterized *Pst* DC3000 strain also fluoresced under these conditions. To assess whether the isolates cause speck disease, bacterial suspensions (10^4^ CFU/mL) of each isolate were vacuum-infiltrated into RG-PtoR and RG-PtoS plants with DC3000 used as a race 0 control strain. RG-PtoR, but not RG-PtoS, plants express a functional Pto/Prf pathway and are resistant to race 0 strains, but not to race 1 strains of *Pst*. We observed that RG-PtoS plants inoculated with strains NC-C3, NC-W201, or DC3000 developed extensive signs of speck disease; however, no disease developed on RG-PtoR plants (**Figure 1A** and Table S1). Based on these observations, NC-C3 and NC-W201 appeared to be *Pst* race 0 strains.

To examine whether the North Carolina isolates produced the virulence-promoting toxin coronatine, we induced production of this toxin before spotting bacterial suspensions on RG-PtoS (see Methods). Both NC-C3 and NC-W201 caused a necrotic spreading lesion surrounded by a diffuse yellow chlorosis suggesting they produce coronatine similar to the coronatine-producing control DC3000 on susceptible RG-PtoS leaves (**Figure 1B**). A DC3000 strain that has a deletion abolishing its coronatine production was used as a negative control (CUCPB5502, A. Collmer, unpublished) and it did not cause chlorosis. Successful PCR amplification of a fragment from the *cfa7* gene from the coronatine biosynthetic gene cluster further suggests that NC-C3 and NC-W201 produce coronatine (**Figure S1**)

### Identification and analysis of the *avrPto* and *avrPtoB* genes in the North Carolina isolates

Race 0 *Pst* strains are distinguished from race 1 strains by the presence of the effector genes *avrPto* or *avrPtoB* whose proteins are recognized by the Pto kinase. Although race 1 strains do not have *avrPto*, they sometimes have an *avrPtoB* homolog, but its protein does not appear to accumulate in these strains and they are therefore not recognized by *Pto*-expressing tomato lines [31], [37]. To test for the presence of *avrPto* and *avrPtoB*, we used PCR to amplify their sequences from isolates NC-C3 and NC-W201 and sequenced the resulting products (**Table S2**). A multiple sequence alignment of the amino acid sequences predicted from the *avrPto* genes in these two isolates revealed their proteins are identical to AvrPto in *Pst* JL1065 (a race 0 strain), which differs from DC3000 AvrPto by 4 amino acids, and less closely related to AvrPto in *P. s.* pv. *syringae* strain B728a (**Figure 1C** and **Figure S2**). The predicted *avrPtoB* (*hopAB*) genes in NC-C3 and NC-W201 were found to encode proteins most similar to the AvrPtoB homolog in T1, but also closely related to that in JL1065, a race 0 strain. The NC-C3 and NC-W201 AvrPtoB homologs were most dissimilar to that in DC3000 (race 0) and in B728a (Figure 1C). Together, the presence of these genes and the disease assays indicate that NC-C3 and NC-W201 are race 0 *Pst* strains.

### Placement of strains within the *P. syringae* phylogeny

The T1-like clade of *Pst* strains identified by Yan et al. (2008) [38] can be distinguished from DC3000 and related *Pst* and *P. s.* pv. *maculicola* strains by the presence of effector genes *hopW1* and *avrA* [11], [39]. We therefore used primers to amplify fragments of these effector genes and PCR products of the expected sizes were identified in NC-C3 and NC-W201 as well as from race 1 *Pst* strain T1 (**Figure S3**), but not in DC3000 as expected. These results, together with the presence of an *avrPto* variant identical to that in JL1065 supports placement of NC-C3 and NC-W201 among T1-like race 0 strains of *Pst* [11], [31].

### Characterization of flagellin in the North Carolina isolates

Peptide regions within the flagellin protein, referred to as flg22 and flgII-28, are major MAMPs that are recognized by plants thereby activating an immune response [3], [11], [40]. *Pst* strains can differ in their FliC sequence and, in some cases, polymorphisms in the regions encoding the MAMPs may allow strains to evade detection by the plant [11]. We therefore investigated whether any polymorphisms occur in the FliC protein expressed by the North Carolina isolates. We PCR-amplified and sequenced fragments of the *fliC* gene from NC-C3 and NC-W201 to obtain the sequence over the flg22 and flgII-28 regions (**Figure S4**). An alignment of the amino acid sequences of these FliC proteins with those from other *Pst* strains revealed 100% conservation in the flg22 region (Figure S3). However, a notable polymorphism was observed in the flgII-28 region. Specifically, both NC-C3 and NC-W201 have a phenylalanine (F) at position 99 that in most other *Pst* strains is a serine (S) (Figure S3). This substitution has been observed previously in the *Pst* race 0 strains K40 and LNPV 17.41, and was shown to decrease the ability of flgII-28 to elicit the production of reactive oxygen species associated with pattern-triggered immunity [11]. This alteration may therefore have evolved to partially evade recognition by a plant PRR protein. Additional polymorphisms were found at positions 106, 110, and 111 where NC-C3 and NC-W201 differ with respect to DC3000 but have the same amino acids as several other *Pst* strains (Figure S4). A summary comparing features of NC-C3 and NC-W201 with several other well-characterized *Pst* strains is presented in **Table S3**.

### Response of heirloom tomatoes to bacterial speck disease in the field

With the availability of local *Pst* isolates we initiated a screen of 13 heirloom tomato varieties (**Table 1**). A first consideration was whether any of the heirloom lines carry the *Pto* gene that would make them resistant to the North Carolina isolates. A PCR-based assay for *Pto* confirmed its presence in the control cultivar RG-PtoR and its absence in RG-PtoS (Table 1). Using this assay we determined that none of the heirloom lines carry the *Pto* gene (Table 1 and **Figure S5**).

**Table 1:** Tomato heirloom varieties used to screen for MAMP responsiveness and resistance to bacterial speck caused by NC-C3, a *Pseudomonas syringae pv. tomato* race 0 isolate.

Tomato accession^a^	Origin	*Pto* gene^b^
Rio Grande-PtoR (RG-PtoR)	Peto Seed Co.,	+
Rio Grande-PtoS (RG-PtoS)	Peto Seed Co.,	−
Moneymaker/LA2706	Netherlands	−
Ailsa Craig/LA2838A/PI262995	England	−
Aker's West Virginia	Unknown	−
Amishpaste	United States	−
Black from Tula	Unknown	−
Brandywine (Sudduth/Quisenberry)	United States	−
Cherokee Purple	United States	−
Favorite/PI636262	United States	−
Orange Strawberry	Unknown	−
Oxheart/NSL193993	United States	−
Rutgers/LA1090	United States	−
Stupice/PI250436	Czechoslovakia	−
Yellow Pear	Unknown	−
Yellow Stuffer	Unknown	−

Tomato cultivar Rio Grande-PtoR containing the *Pto* locus was used as a resistant control. Rio Grande-PtoS, and Moneymaker were used as susceptible controls.

a See Male et al. (1999) and Goldman (2008) for additional information about each accession.

b A PCR-based assay was used to determine if the *Pto* resistance gene is present (+) or absent (−).

**Figure 2:**
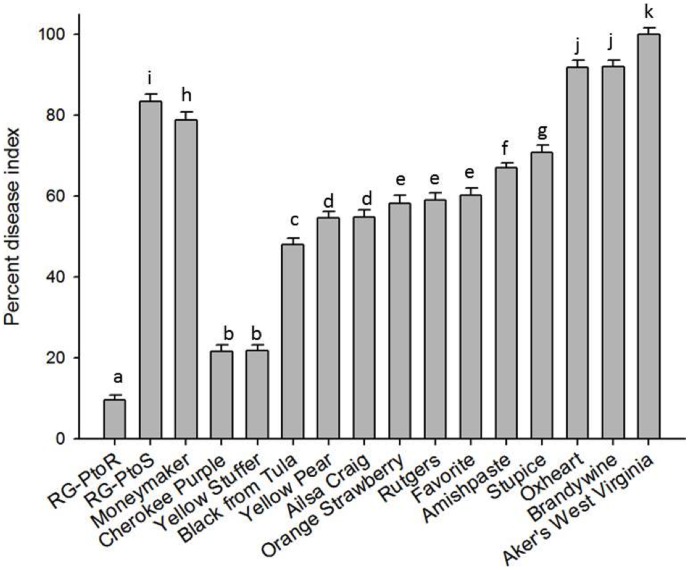
Response of heirloom tomatoes to NC-C3 in the field. Disease severity of heirloom tomatoes inoculated with race 0 *Pst* isolate NC-C3 under field conditions (summer 2012). RG-PtoR has the *Pto* gene and was included as a resistant control. RG-PtoS and Moneymaker lack *Pto* and were included as susceptible controls. Inoculated plants were scored at 7 dpi using a 0–5 scale as described in Materials and Methods. Mean percent disease index (PDI; see Methods) was calculated from the disease scores of each genotype by totaling the score of 18 plants (three replicates each of 6 plants per line) and expressing the value as a percentage (%). Means marked with the same letter are not statistically different at a 5% probability level based on least significant difference separation.

The 13 heirloom lines along with controls were planted in the field in the summer of 2012 using a random complete block design (see Methods). Four weeks after transplanting in the field, the plants were inoculated with a 10^7^–10^8^ CFU/mL of NC-C3 using a hand pump sprayer. Signs of bacterial speck disease began to appear 3 days after inoculation and ranged from severe (similar or greater disease as visible on the susceptible cultivars RG-PtoS and Moneymaker) to mild. A disease index was used to score the plant responses and photographs were taken of representative plants (**Figure 2** and **Figure S6**). None of the heirloom varieties were as speck-resistant as *Pto*-expressing RG-PtoR although Cherokee Purple and Yellow Stuffer developed very little signs of the disease. These observations indicate there is substantial natural variation in heirloom tomatoes for response to this local *Pst* strain.

### Responses of heirloom tomatoes to three MAMPs

**Figure 3:**
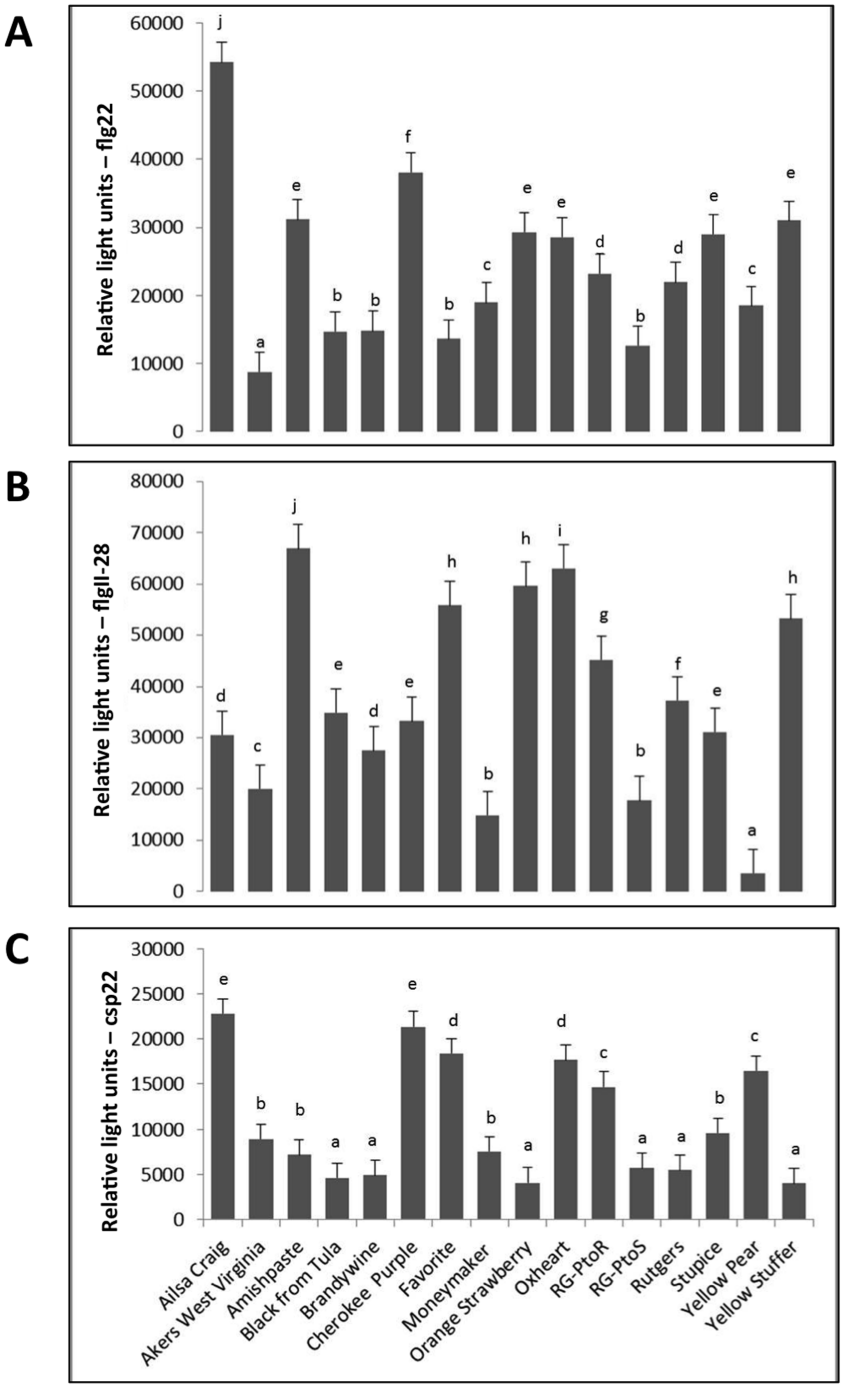
Natural variation in heirloom tomatoes for reactive oxygen species (ROS) production upon exposure to flg22, flgII-28, and csp22. **A**) ROS production was measured over a period of 30 minutes in leaves from 4-week old heirloom tomatoes after exposure to 100 nM flg22. **B**) ROS production was measured over a period of 40 minutes in leaves from 4-week old heirloom tomatoes after exposure to 100 nM flgII-28. **C**) ROS production was measured over a period of 20 minutes in 4-week old heirloom tomato leaves after exposure to 500 nM csp22. The data are shown as mean ± SE from 8 leaf discs analyzed in triplicate with the experiments being repeated three times. Means marked with the same letter are not statistically different at a 5% probability level based on least significant difference separation.

We next investigated whether the heirloom varieties exhibited natural variation in their response to three bacterial-derived MAMP peptides, flg22, flgII-28, and csp22. Leaf discs from each tomato line were treated with the individual peptides and processed in an assay to measure reactive oxygen species (ROS). The response of each line was plotted revealing a broad range of responses to each MAMP (**Figure 3**). Two heirlooms, Cherokee Purple and Ailsa Craig, responded strongly to all three MAMPs. The composite response to all three MAMPs was the lowest for four heirlooms: Aker's West Virginia, Brandywine, Black from Tula, and Yellow Pear. We could not discern a clear correlation from the lines between MAMP responsiveness and the disease score derived from the field experiment. However, it is notable that Cherokee Purple had the lowest disease index and the highest overall ranking for MAMP responsiveness whereas Aker's West Virginia and Brandywine had high disease indexes and some of the lowest rankings for MAMP responsiveness. However, there were several counter-examples, with Oxheart, for example, responding relatively strongly to MAMPs but being highly susceptible to speck disease in the field. An unexpected result was the different MAMP-responsiveness observed between RG-PtoR and RG-PtoS. These lines, which are near-isogenic and should differ primarily at the *Pto* locus, were expected to respond similarly to the MAMPs. RG-PtoR, however, responded much stronger to all three MAMPs as compared with RG-PtoS (Figure 3). A summary of the response of each heirloom to *Pst* and the three MAMPs is presented in **Table S4**.

### A genetic approach to characterizing MAMP responsiveness

**Figure 4:**
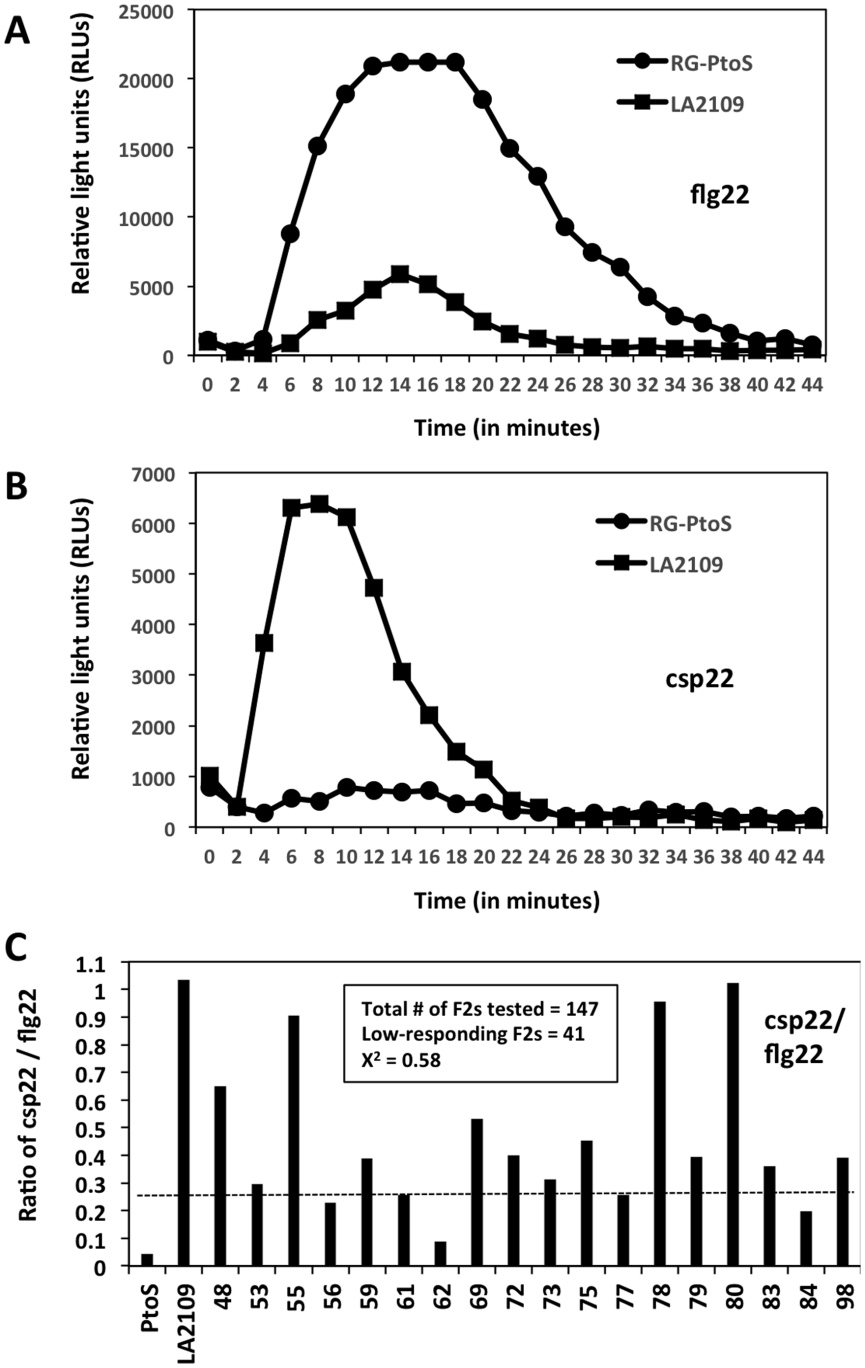
A genetic approach to characterize csp22 responsiveness. Leaf discs from RG-PtoS and LA2109 were treated with 100 nM flg22 (**A**) or 1 µM csp22 (**B**) and ROS production measured every 2 minutes over the time course shown. The curves are the average of 2 plants from independent experiments, and are representative of the trend observed with several other plants. **C**) To identify csp22 low-responding F_2_ plants, the sum of the ROS curves (as shown in A) was determined for flg22 and csp22, and the ratio of csp22-to-flg22 response was determined (csp22/flg22). The numbers at the bottom indicate individual F2 plants. A threshold value of 0.25 was set as the upper limit for defining low responsiveness. Of 147 F_2_ plants tested, 41 were classified as low csp22 responders. A chi-square test for fitting to a 3∶1 was performed and Χ^2^ = 0.58.

The discovery of natural variation for MAMP responsiveness among tomato lines presents the opportunity to identify the underlying loci. Towards this goal, we crossed two lines, RG-PtoS and LA2109, which respond differently to csp22, and developed an F_2_ population. RG-PtoS has a very weak response to csp22 whereas LA2109, a *Solanum habrochaites* accession, responds strongly; both respond to flg22 although LA2109 much less than RG-PtoS (**Figure 4AB**). We chose LA2109, a wild species accession, as a parent for these experiments because the DNA polymorphism in this species compared to RG-PtoS will facilitate a future mapping-by-sequencing approach. We observed that F_1_ plants from this cross exhibited csp22 responsiveness similar to LA2109 (data not shown) indicating the locus displays dominant gene action.

Because RG-PtoS and LA2109 respond differently to flg22, we tested individual F2 plants for their response to both peptides and plotted the ratio of their csp22-to-flg22 response (**Figure 4C**). An initial screen of 147 F_2_ plants derived from a single F_1_ plant identified 41 plants that showed a weak response to csp22. This conforms to a 3∶1 ratio (X^2^ = 0.58) indicating the csp22 responsiveness is segregating as a single locus. We refer to this locus as *Csr1*, csp22-responsive locus 1. These experiments indicate that the natural variation for MAMP responsiveness should be amenable to map-based cloning of the underlying loci.

## Discussion

We have discovered that significant natural variation exists in heirloom tomato lines for their response to *Pst* and three bacterial peptide MAMPs. This work necessitated the identification of a locally-occurring natural isolate of *Pst* in order to avoid release of a non-native strain of this pathogen into an important tomato growing region of North Carolina. We were successful in isolating two local strains of *Pst* both of which appear highly similar and may in fact be identical. One of these strains was used in field studies and revealed a wide range of responses to bacterial speck disease among the heirlooms with a few lines showing relatively strong basal resistance. Similarly, by using a simple ROS assay we discovered a range of responses in the heirlooms to bacterial MAMPs. Together, these results demonstrate that heirloom tomato lines may be a useful source of natural variation for breeding basal resistance into fresh market tomatoes and they can be used as parents in mapping populations to identify the loci responsible for these differential responses.

The bacterium *Pst* has been reported in nearly all temperate regions of the world [41], [42]. Serious outbreaks have occurred in eastern Canada, USA and Israel and, in some cases, have resulted in economically significant crop losses [31], [33], [43], [44]. The *Pto* resistance gene is a highly effective source of resistance to race 0 strains, but the spread of race 1 strains is a source of concern. An early report of race 1 strains in Canada [41] was followed by a report from Bulgaria [45]. Recently, the occurrence of race 1 in tomato has been reported in Serbia [46], Portugal [47] and California [31], [33]. A previous study reported the incidence of bacterial speck of tomato in western North Carolina and the *Pst* strains involved were characterized as copper resistant and streptomycin sensitive [48], but no investigation of the *Pst* phylogeny or race characterization was conducted. In the present study, two isolates from North Carolina, NC-C3 and NC-W201, were characterized and found to belong to the T1-like clade. The presence of *avrPto* and *avrPtoB* genes in these isolates and their lack of pathogenicity on RG-PtoR confirmed them to be race 0 strains.

Both of the North Carolina isolates were found to have a phenylalanine at position 99 (F99) in the flgII-28 region of their flagellin protein. This is in common with *Pst* strains such as K40, LNPV17.41, IPV-CT28.31 and LNPV17.41 but differs from many other *Pst* strains which have a serine at this position [11]. The S99F substitution reduces the ability of flgII-28 to elicit the production of ROS and might have evolved as a way for the pathogen to attenuate the host response to this MAMP. Overall, for the features we investigated, NC-C3 and NC-W201 are identical to K40 which was collected from tomatoes in the USA in 2005 [11]. All three of these strains have *avrPto*, *avrPtoB*, *avrA* and *hopW1*, produce coronatine, and have two substitutions in the flgII-28, A96 and F99. Future sequencing of the genomes of NC-C3 and NC-W201 will be needed to comprehensively compare them with sequenced *Pst* strains (http://pseudomonas-syringae.org/).

We observed significant variation in the response of the heirlooms to bacterial speck disease. Two lines, Cherokee Purple and Yellow Stuffer, developed very few necrotic spots or chlorosis after spray inoculation and might be a useful source of basal resistance to speck disease (neither one has the *Pto* gene). Most of the heirlooms, however, developed moderate to severe signs of bacterial speck disease. Although some of these lines might still be good sources of loci for MAMP-responsiveness (see below) their primary use for future work could be as parents in a mapping population with Cherokee Purple or Yellow Stuffer in order to dissect the genetic basis for the speck tolerance in these two lines.

There was also a range of significant variation for the response of the heirloom lines to the three peptide MAMPs. Interestingly, Cherokee Purple was again one of the notable lines with a very strong response to all three MAMPs. Other lines that had a high overall score for MAMP-responsiveness were Ailsa Craig, RG-PtoR, and Oxheart (Table S4). Three lines showed very weak responses in the ROS assay to two of the MAMPs: Black from Tula, Brandywine, and Yellow Pear. As is evident from Table S4, there was not a strong correlation between the response to *Pst* and the response to various MAMPs. This is perhaps not surprising as there are probably many plant immune (and susceptibility) factors and *Pst* virulence determinants involved in the host response to speck disease. Nevertheless, it is noticeable that many of the lines that showed a poor overall response to MAMPs also were among the most susceptible to speck disease (Table S4) and it seems likely that MAMP detection does play a role in the field in providing basal defense against *Pst*.

There are several possibilities that might account for the different degrees of responsiveness to flg22, flgII-28 and csp22 that we observed. First, the PRRs in these lines might have polymorphisms that decrease or increase their affinity for binding the peptides, or a specific PRR might be missing from certain heirlooms. Second, the PRR may bind a specific MAMP effectively, but have a polymorphism in an intracellular domain that decreases its ability to activate signal transduction. Third, specific heirlooms may have polymorphisms in or lack certain downstream components that are required for signaling or for the generation of ROS. It has been reported recently that extensive variation for flg22 perception occurs in *A. thaliana* accessions and its Brassicaceae relatives which correlates with the severity of elicited defense responses and bacterial multiplication [49]. This variation in flg22 perception resulted from amino acid substitutions in the FLS2 receptor, FLS2 protein abundance, or changes in abundance of components common to pathways downstream of MAMP perception, all of which are likely to contribute to the quantitative variation in the Brassicaceae family. In future work these possibilities could also be investigated among the heirloom lines for FLS2 and eventually for the PRRs and pathways that respond to flgII-28 and csp22 when they are characterized.

It was unexpected that RG-PtoR and RG-PtoS showed significant differences in their response to all three MAMPs. RG-PtoR was derived from multiple backcrosses to RG-PtoS in order to retain the *Pto/Prf* locus on chromosome 5 and yet restore other horticulturally-important traits in the RG-PtoS line [27]. These near-isogenic lines are expected to have >95% of the same loci (and to differ primarily at the *Pto* locus) and would be anticipated to respond similarly to the three MAMPs. In fact, both RG-PtoR and RG-PtoS are similarly susceptible to race 1 strains of *Pst*. The differential response of these lines to flg22, flgII-28 and csp22 raises three possibilities. First, *Pto* or a *Pto* family member could play a role in MAMP responsiveness. This seems unlikely as we have not observed a difference in MAMP responsiveness between RG-PtoR and a RG-PtoR line that has knocked down expression of *Pto* and two *Pto* family members ([50], G. Martin, unpublished). It is possible that the *Prf* allele in this line contributes to a stronger MAMP response as compared to the allele in RG-PtoS. Second, another MAMP-responsive locus (or loci) may exist close to the *Pto/Prf* locus and has been carried along by linkage drag despite the numerous cycles of backcrosses. Finally, another locus (or loci) that confers MAMP responsiveness might reside on another chromosomal region in RG-PtoR. This region might have been carried along during the backcrossing process because it makes a substantial contribution to speck resistance. We are currently analyzing an F_2_ population derived from an RG-PtoR/RG-PtoS cross in order to investigate these possibilities.

Our finding of natural variation in the heirlooms for MAMP responsiveness lays the foundation for using a genetic approach to determine the underlying mechanisms for differences in MAMP responsiveness. Towards this goal, we initiated a project to map the locus (or loci) responsible for csp22 detection or response by crossing RG-PtoS (a low responder to csp22) and a wild relative of tomato, *Solanum habrochaites*, that we had observed in another project to be highly responsive to csp22. F_2_ plants derived from this cross showed a differential response to csp22 in a ratio close to a 3∶1, indicating a single Mendelian locus is involved. In the future, bulked segregant analysis combined with next-generation sequencing of the pools should allow mapping of the csp22 responsive locus, which we refer to *Csr1*. Refinement of the map position of *Csr1* will allow marker-based introgression of the locus into breeding lines and a test of whether it improves basal resistance to bacterial disease in the field. High-resolution mapping may eventually lead to cloning of the locus and investigation of how it confers enhanced resistance to csp22.

## Materials and Methods

### Sample collection, isolation and culture of bacteria

Bacterial strains were collected in 2011 from naturally-infected tomato leaves in western North Carolina. One isolate, NC-C3, was collected from the farm of Mr. Kirby Johnson, Mills River, Henderson County (latitude: 35.3801180 and longitude: −82.5625760). The other, NC-W201, was collected from the farm of Mr. Kent Cochran, Whittier, Jackson County (latitude: 35.4066632 and longitude: −83.3277315). These locations are on private land and permission was obtained from the owners for the collections. Leaves showing speck-like disease symptoms were surface-sterilized, and five leaf disks were placed into a 1.5 ml tube with 1 mL of sterile water and incubated at room temperature for 30 min. The tubes were processed in a bullet blender (Next Advance, NY, USA) for 30 sec and a portion of the extract was plated on solid King's medium B (KB). Levan-forming colonies which fluoresced under UV light on KB were selected and transferred to nutrient agar (BD Difco, Sparks, MD, USA) amended with 2% glycerol.

### DNA extraction from bacteria and plants

Bacterial isolates were grown on solid KB medium for 24–48 h at 28°C prior to DNA extraction. DNA was resuspended in sterile distilled water, quantified with a Nanodrop 2000 spectrophotometer (Thermo Scientific, Wilmington, Delaware, USA) and diluted to 50 ng/µl for use in PCR assays. Genomic DNA was isolated from tomato leaves using a Cetyl trimethyl ammonium bromide (CTAB) based method [51].

### PCR amplification, DNA sequencing and sequence analysis

PCRs were performed using Taq DNA polymerase (NEB, Biolabs Inc., Ipswich, MA, USA) for all amplifications except for the purpose of sequencing *avrPto* and *avrPtoB* in which case Phusion High Fidelity DNA polymerase (Fisher Scientific, Pittsburgh, PA, USA) was used. Primers and PCR conditions are described in Table S2. PCR products were processed using the PCR cleanup DNA purification kit (Sigma-Aldrich, St. Louis, MO, USA) before sequencing. All sequences were evaluated using BLAST software (http://www.ncbi.nlm.nih.gov/BLAST/) and multiple-sequence alignments were generated using ClustalW software (http://www.ebi.ac.uk/Tools/msa/clustalw2/).

### Plant growth conditions, inoculation methods, disease and coronatine assessment

Seeds were sown in a soil bed containing peat and perlite. After two weeks, seedlings were planted in 72-cell flats (56×28 cm^2^) in potting mix in the first week of May and transplants, at about 6 weeks from seed were planted by hand in the field. Six plants of each cultivar were planted with 45 cm between plants in the row, and 150 cm row-to-row spacing. The soil was a clay-loam and the natural day light photoperiod was about 14/10 hr with 25–30°C high and 14–16°C low temperatures. In field experiments, three replicates were used for each cultivar in a randomized complete block design. Plants were inoculated 4 weeks after transplanting in the field.

Bacterial inoculum was prepared by growing *Pst* strains on KB plates at 28°C for 24–48 h. Bacterial cells were suspended in 10 mM MgCl_2_ and the suspensions were adjusted to 10^7^–10^8^ colony-forming units per mL (CFU/mL) using a hand-pump sprayer (Solo, Oesco Inc., Conway, MA, USA).

Speck symptoms appeared 3 to 7 days post inoculation (dpi). Disease assessments were made 7 dpi and scored according to Chambers and Merriman [52] with slight modifications. Briefly, the disease was scored using a scale of 0–5, with 0 = no disease and 5 = severe disease. Percent disease index (PDI) was calculated based on the following formula: PDI (%) = (Mean value of disease score observed in cultivar)/(Maximum disease score observed in cultivars)×100.

For the coronatine assay, strains were grown on mannitol-glutamate (MG) solid and liquid media containing mannitol (10 g/l), L-glutamic acid (2 g/l), KH_2_PO_4_ (0.5 g/liter), NaCl (0.2 g/liter), MgSO_4._7 H_2_O (0.2 g/liter), Agar (1.8%). The pH of the media was adjusted to 7.0 with 1N NaOH prior to autoclaving. On the day of the experiment, the bacteria were suspended in MG and 50 µM ferric citrate and adjusted the OD_600_ to 0.1. The bacterial suspension was diluted to different ratio (1∶5, 1∶10, 1∶20, 1∶50, and 1∶100) and 20 µl of inoculum was spotted onto wounded leaves. Chlorosis associated with coronatine production was observed on leaves within 3–5days.

### Peptides

Peptides were synthesized at >90% purity by EZ Biolab (Carmel, IN, USA). The sequence of flg22 (QRLSTGSRINSAKDDAAGLQIA) was described by Felix et al. (10). The sequence corresponding to the *Pst* DC3000 variant of flgII-28 (ESTNILQRMRELAVQSRNDSNSATDREA) was described by Cai et al. (2011) [11]. The sequence of csp22 (AVGTVKWFNAEKGFGFITPDDG) was described by Felix and Boller [13]. Peptides were suspended in sterile water to a concentration of 1 µM and used at the dilutions stated in the figure legends.

### ROS assay

The production of reactive oxygen species (ROS) was measured as described previously [53]. Eight 2 mm discs were excised from leaves of 3–4 week-old tomato plants grown in the greenhouse and luminescence was measured in a Glomax 96 microplate luminometer (Promega, Madison, WI, USA) at 2 min intervals for 20–50 min after the addition of the test solution.

### Data analysis

Analysis of variance (ANOVA) was performed using SAS Software to determine the differences between the tomato lines for MAMP responsiveness. Least square means for MAMP response of each cultivar was determined and compared using least significant difference (LSD) value at the 5% probability level.

## Supporting Information

Figure S1 **The North Carolina isolates have the** ***cfa7*** **gene, which lies in the coronatine biosynthetic gene cluster.** A PCR assay was used to amplify a region (689 bp) within the *cfa7* coronatine biosynthetic gene from *Pseudomonas syringae* isolates from NC-C3 and NC-W201. M, molecular marker of 100-bp fragments (NEB, Biolabs Inc., Ipswich, MA, USA). (−) indicates a negative control.
(PPTX)

Figure S2 **Amino acid alignment of the AvrPto and AvrPtoB proteins in the North Carolina isolates and other** ***P. syringae*** **strains.** **A**) Alignment of the AvrPto amino acid sequence from the North Carolina isolates and other *P. syringae* strains. The N- and C-termini of AvrPto from isolates NC-C3 and NC-W201 were not determined. The sequences of the two field isolates are shown as one sequence because they are identical. The Genbank accession numbers are YP237724 (AvrPto_B728a_), L20425 (AvrPto_JL1065_), NP793764 (AvrPto_DC3000_), KC986841(AvrPto_NC-C3_) and KC986842 (AvrPto_NC-W201_). **B**) Alignment of the AvrPtoB amino acid sequence from the North Carolina isolates and other *P. syringae* strains. To obtain the 5′ and 3′ regions of the *avrPtoB* gene from NC-C3 and NC-W201, additional primers were designed based on the T1 *avrPtoB* sequence. The Genbank accessions numbers are YP237724 (avrPtoB_B578a_), DQ133535 (avrPtoB_JL1065_), ZP03398509 (avrPtoB_T1_), NP792881 (avrPtoB_DC3000_), KC986843 (AvrPtoB_NC-C3_) and KC986844 AvrPtoB_NC-W201_). AvrPphF (AAF67149) from *P. s.* pv. *phaseolicola* was used as an outlier. Alignments were developed using Muscle and then imported into Genedoc for shading of consensus residues. Black represents amino acids that are identical in all sequences, light and dark grey indicate residues of lesser conservation, and white represents a divergent residue.
(PPTX)

Figure S3 **Determining whether the North Carolina** ***P. s.*** **pv.** ***tomato*** **isolates have effector genes** ***hopW1*** **and** ***avrA*** **.** **A**) Primers for *hopW1* were used to amplify a 1,480 base pair fragment from DNA of the North Carolina isolates NC-C3 and NC-W201 or *Pst* strains DC3000 and T1. **B**) Primers for *avrA* were used to amplify a 1,030 base pair fragment from the same DNA samples. Details of the primers and reaction conditions are provided in Table S2.
(PPTX)

Figure S4 **MAMP regions of the FliC proteins from North Carolina isolates NC-C3 and NC-W201 and other** ***P. s.*** **pv.** ***tomato*** **strains.** Primers for *fliC* were used to amplify an 849-bp fragment of the gene from DNA of the North Carolina isolates or *Pst* strain DC3000. The *fliC* fragments were sequenced from NC-C3 and NC-W201 and the derived amino acid sequences spanning flg22 and flgII-28 were aligned with the corresponding sequences from *Pst* strains DC3000 (GenBank No. AB061231.1), T1 (ZP_03395718.1), LNPV17.41 (JF261012.1); Colombia198 (JF261011.1); Colombia338 (JF261013.1) and *P. syringae* pv. *maculicola* ES4326. The asterisk (*) indicates an amino acid difference present in *Pst* strains Colombia 198 and Colombia 338. The red letters indicate other important amino acid differences among the strains.
(PPTX)

Figure S5 **The heirloom lines do not have the** ***Pto*** **gene.** PCR products diagnostic for resistant or susceptible *Pto* haplotypes were amplified from genomic DNA and digested with FokI. The product at about 900 bp (red arrow) is diagnostic of the *Pto* gene (present in RG-PtoR and Ontario 7710). The product at about 250 bp (black arrow) is diagnostic of lines lacking *Pto* (such as RG-PtoS and Moneymaker). None of the heirlooms appear to have the *Pto* gene.
(PPTX)

Figure S6 **Heirloom tomato lines showed differential susceptibility to North Carolina isolate NC-C3.** Photos of bacterial speck disease on plants from the field inoculation experiment using North Carolina isolate NC-C3. Photographs were taken on the 7th day after inoculation. Red arrows point to signs of the disease.
(PPTX)

Table S1 **Bacterial isolates from western North Carolina compared with** ***P. s.*** **pv.** ***tomato*** **DC3000.**
(DOCX)

Table S2 **DNA primers and PCR conditions used in this study.**
(DOCX)

Table S3 **Comparison of features of the North Carolina isolates with other well-characterized** ***P. s.*** **pv.** ***tomato*** **strains.**
(DOCX)

Table S4 **Summary of responses to MAMPs and speck disease in the field.**
(DOCX)
