# Supplementary material for: Natural Variation for Responsiveness to flg22, flgII-28, and csp22 and Pseudomonas syringae pv. tomato in Heirloom Tomatoes
Source: PLoS One. 2014 Sep 2;9(9):e106119. doi: 10.1371/journal.pone.0106119 (PMC4152135; doi:10.1371/journal.pone.0106119)
Supplement: Figure S1 — The North Carolina isolates have the cfa7 gene, which lies in the coronatine biosynthetic gene cluster. A PCR assay was used to amplify a region (689 bp) within the cfa7 coronatine biosynthetic gene from Pseudomonas syringae isolates from NC-C3 and NC-W201. M, molecular marker of 100-bp fragments (NEB, Biolabs Inc., Ipswich, MA, USA). (−) indicates a negative control. (PPTX) [file pone.0106119.s001.pptx]

## Slide 1
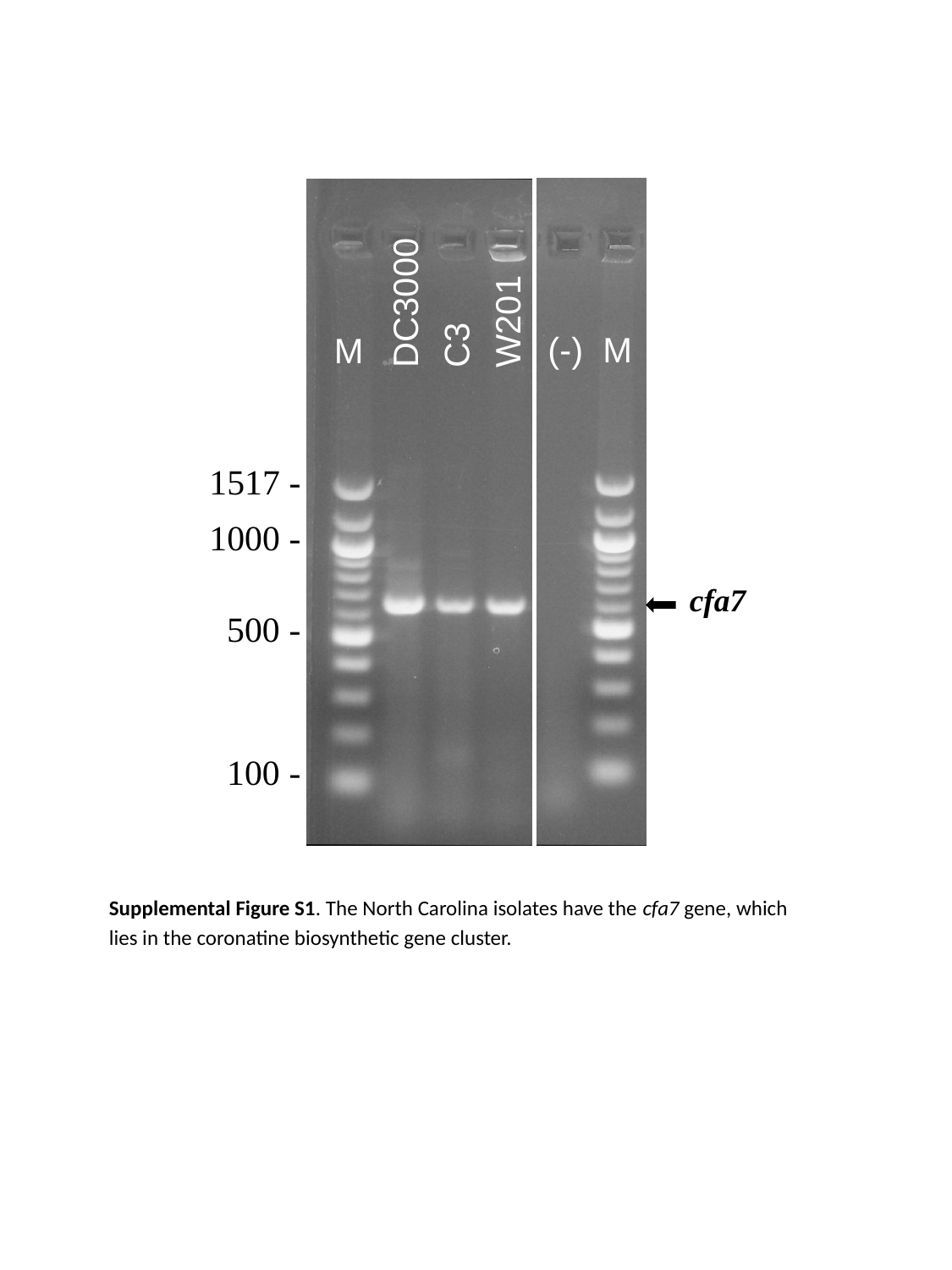

DC3000
C3
W201
(-) M
M
1517 -
1000 -
cfa7
500 -
100 -
Supplemental Figure S1. The North Carolina isolates have the cfa7 gene, which lies in the coronatine biosynthetic gene cluster.
