# Supplementary material for: Natural Variation for Responsiveness to flg22, flgII-28, and csp22 and Pseudomonas syringae pv. tomato in Heirloom Tomatoes
Source: PLoS One. 2014 Sep 2;9(9):e106119. doi: 10.1371/journal.pone.0106119 (PMC4152135; doi:10.1371/journal.pone.0106119)
Supplement: Figure S2 — Amino acid alignment of the AvrPto and AvrPtoB proteins in the North Carolina isolates and other P. syringae strains. A) Alignment of the AvrPto amino acid sequence from the North Carolina isolates and other P. syringae strains. The N- and C-termini of AvrPto from isolates NC-C3 and NC-W201 were not determined. The sequences of the two field isolates are shown as one sequence because they are identical. The Genbank accession numbers are YP237724 (AvrPtoB728a), L20425 (AvrPtoJL1065), NP793764 (AvrPtoDC3000), KC986841(AvrPtoNC-C3) and KC986842 (AvrPtoNC-W201). B) Alignment of the AvrPtoB amino acid sequence from the North Carolina isolates and other P. syringae strains. To obtain the 5′ and 3′ regions of the avrPtoB gene from NC-C3 and NC-W201, additional primers were designed based on the T1 avrPtoB sequence. The Genbank accessions numbers are YP237724 (avrPtoBB578a), DQ133535 (avrPtoBJL1065), ZP03398509 (avrPtoBT1), NP792881 (avrPtoBDC3000), KC986843 (AvrPtoBNC-C3) and KC986844 AvrPtoBNC-W201). AvrPphF (AAF67149) from P. s. pv. phaseolicola was used as an outlier. Alignments were developed using Muscle and then imported into Genedoc for shading of consensus residues. Black represents amino acids that are identical in all sequences, light and dark grey indicate residues of lesser conservation, and white represents a divergent residue. (PPTX) [file pone.0106119.s002.pptx]

## Slide 1
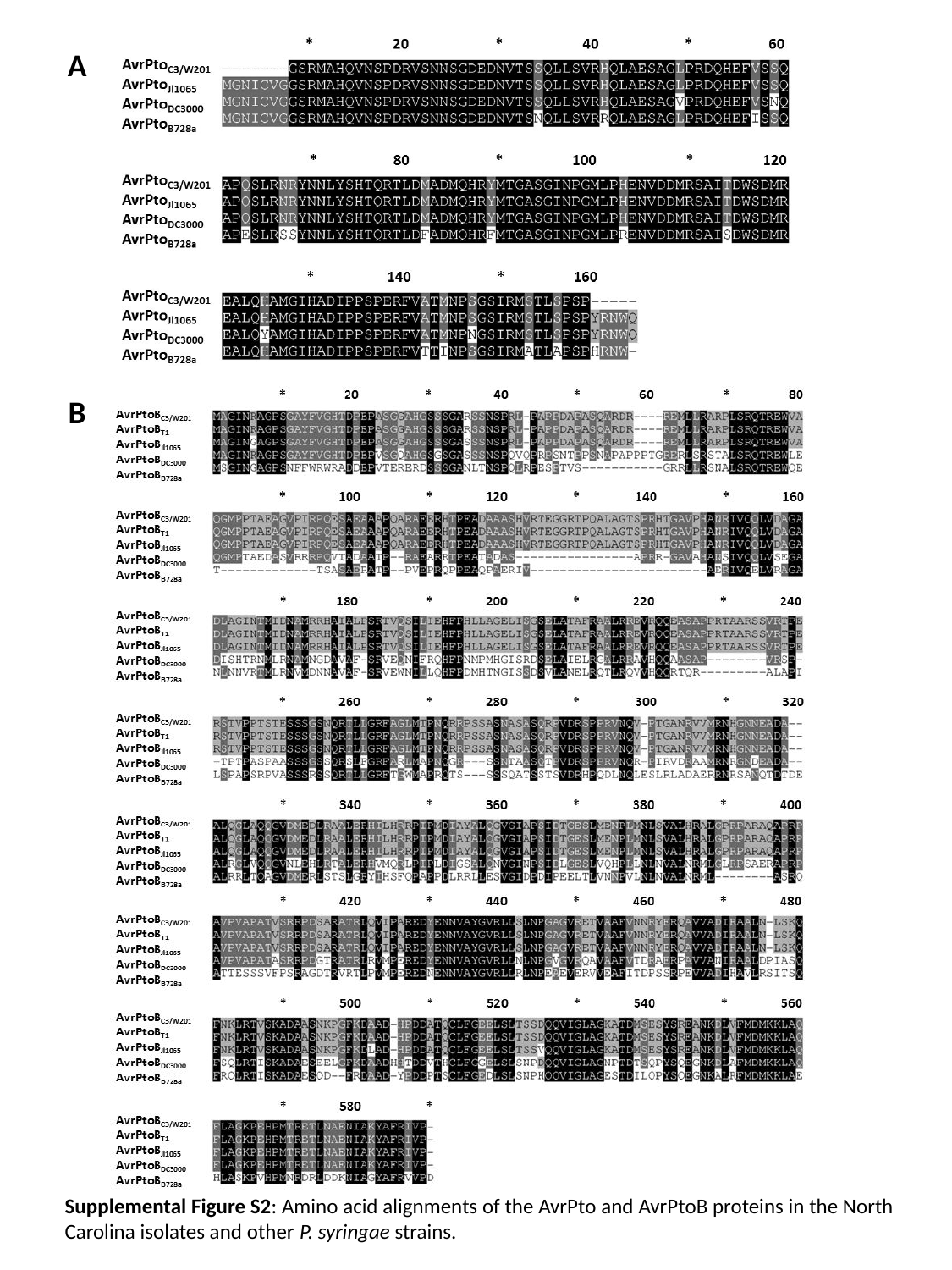

A
B
Supplemental Figure S2: Amino acid alignments of the AvrPto and AvrPtoB proteins in the North Carolina isolates and other P. syringae strains.
