# Supplementary material for: Natural Variation for Responsiveness to flg22, flgII-28, and csp22 and Pseudomonas syringae pv. tomato in Heirloom Tomatoes
Source: PLoS One. 2014 Sep 2;9(9):e106119. doi: 10.1371/journal.pone.0106119 (PMC4152135; doi:10.1371/journal.pone.0106119)
Supplement: Figure S3 — Determining whether the North Carolina P. s. pv. tomato isolates have effector genes hopW1 and avrA . A) Primers for hopW1 were used to amplify a 1,480 base pair fragment from DNA of the North Carolina isolates NC-C3 and NC-W201 or Pst strains DC3000 and T1. B) Primers for avrA were used to amplify a 1,030 base pair fragment from the same DNA samples. Details of the primers and reaction conditions are provided in Table S2. (PPTX) [file pone.0106119.s003.pptx]

## Slide 1
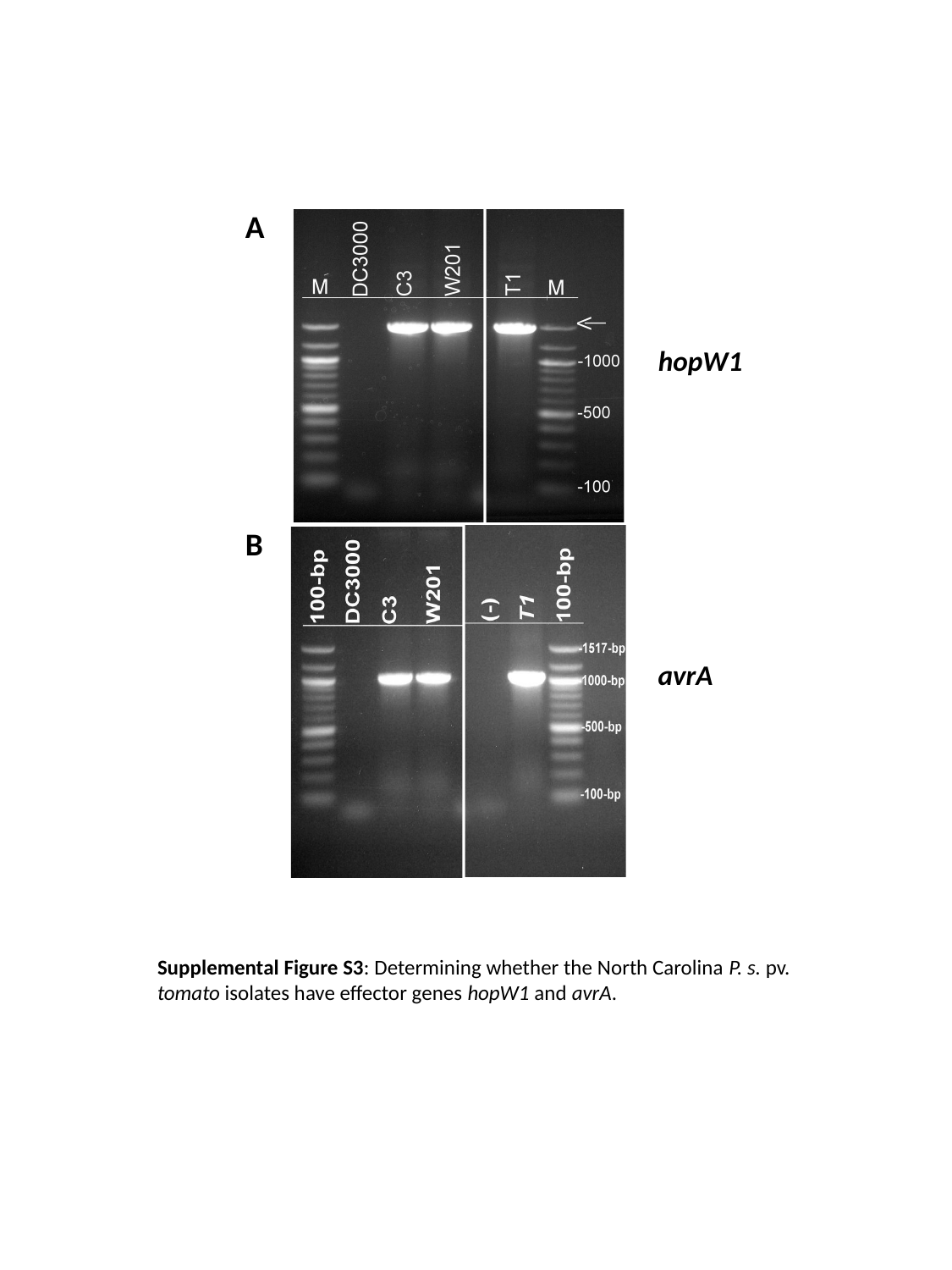

A
hopW1
B
B
avrA
Supplemental Figure S3: Determining whether the North Carolina P. s. pv. tomato isolates have effector genes hopW1 and avrA.
