# Supplementary material for: Natural Variation for Responsiveness to flg22, flgII-28, and csp22 and Pseudomonas syringae pv. tomato in Heirloom Tomatoes
Source: PLoS One. 2014 Sep 2;9(9):e106119. doi: 10.1371/journal.pone.0106119 (PMC4152135; doi:10.1371/journal.pone.0106119)
Supplement: Figure S4 — MAMP regions of the FliC proteins from North Carolina isolates NC-C3 and NC-W201 and other P. s. pv. tomato strains. Primers for fliC were used to amplify an 849-bp fragment of the gene from DNA of the North Carolina isolates or Pst strain DC3000. The fliC fragments were sequenced from NC-C3 and NC-W201 and the derived amino acid sequences spanning flg22 and flgII-28 were aligned with the corresponding sequences from Pst strains DC3000 (GenBank No. AB061231.1), T1 (ZP_03395718.1), LNPV17.41 (JF261012.1); Colombia198 (JF261011.1); Colombia338 (JF261013.1) and P. syringae pv. maculicola ES4326. The asterisk (*) indicates an amino acid difference present in Pst strains Colombia 198 and Colombia 338. The red letters indicate other important amino acid differences among the strains. (PPTX) [file pone.0106119.s004.pptx]

## Slide 1
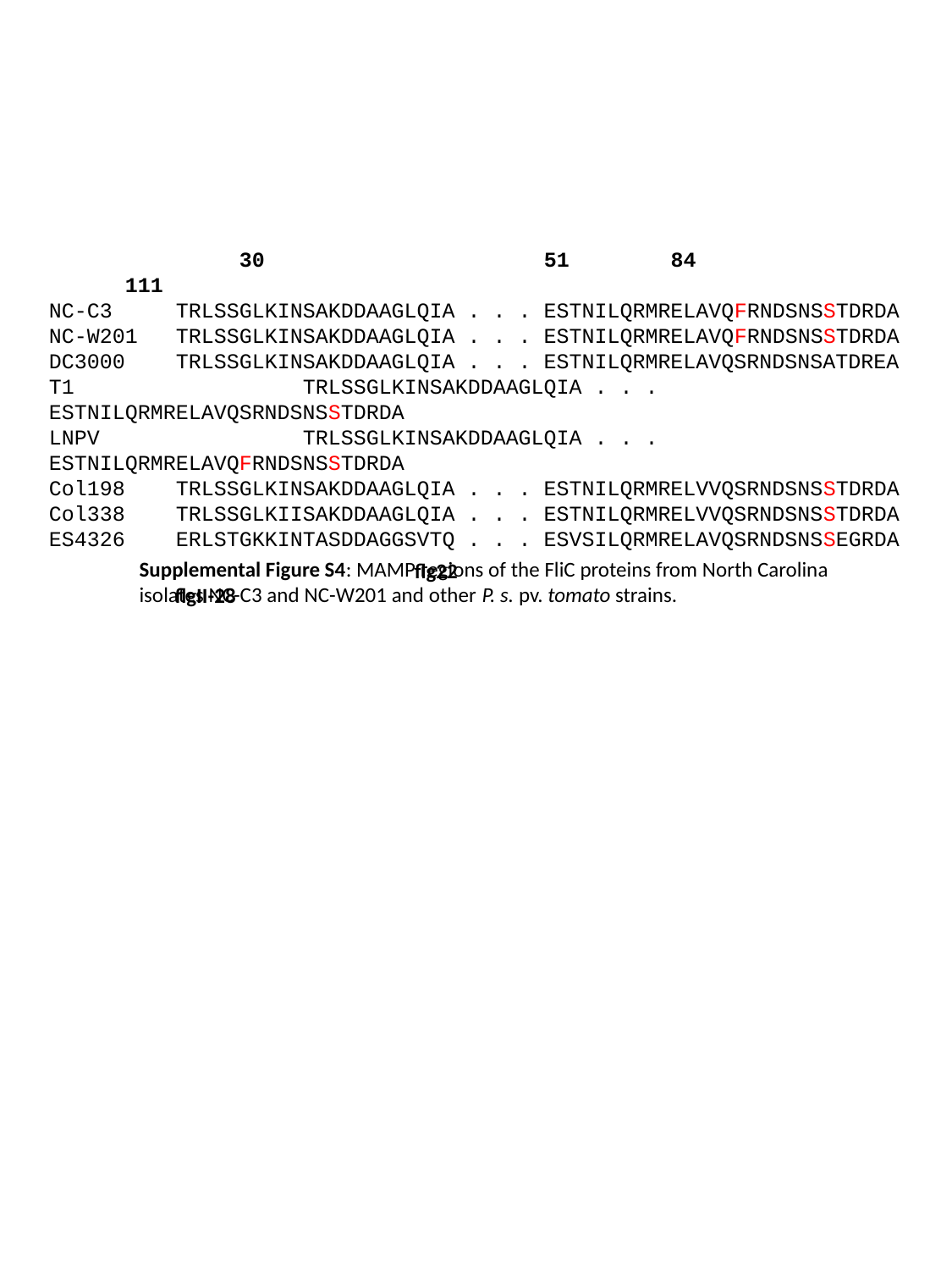

30		 51 84 111
NC-C3	TRLSSGLKINSAKDDAAGLQIA . . . ESTNILQRMRELAVQFRNDSNSSTDRDA
NC-W201	TRLSSGLKINSAKDDAAGLQIA . . . ESTNILQRMRELAVQFRNDSNSSTDRDA
DC3000	TRLSSGLKINSAKDDAAGLQIA . . . ESTNILQRMRELAVQSRNDSNSATDREA
T1		TRLSSGLKINSAKDDAAGLQIA . . . ESTNILQRMRELAVQSRNDSNSSTDRDA
LNPV		TRLSSGLKINSAKDDAAGLQIA . . . ESTNILQRMRELAVQFRNDSNSSTDRDA
Col198	TRLSSGLKINSAKDDAAGLQIA . . . ESTNILQRMRELVVQSRNDSNSSTDRDA
Col338	TRLSSGLKIISAKDDAAGLQIA . . . ESTNILQRMRELVVQSRNDSNSSTDRDA
ES4326	ERLSTGKKINTASDDAGGSVTQ . . . ESVSILQRMRELAVQSRNDSNSSEGRDA
		 flg22			 flgII-28
Supplemental Figure S4: MAMP regions of the FliC proteins from North Carolina isolates NC-C3 and NC-W201 and other P. s. pv. tomato strains.
