# Supplementary material for: Natural Variation for Responsiveness to flg22, flgII-28, and csp22 and Pseudomonas syringae pv. tomato in Heirloom Tomatoes
Source: PLoS One. 2014 Sep 2;9(9):e106119. doi: 10.1371/journal.pone.0106119 (PMC4152135; doi:10.1371/journal.pone.0106119)
Supplement: Figure S5 — The heirloom lines do not have the Pto gene. PCR products diagnostic for resistant or susceptible Pto haplotypes were amplified from genomic DNA and digested with FokI. The product at about 900 bp (red arrow) is diagnostic of the Pto gene (present in RG-PtoR and Ontario 7710). The product at about 250 bp (black arrow) is diagnostic of lines lacking Pto (such as RG-PtoS and Moneymaker). None of the heirlooms appear to have the Pto gene. (PPTX) [file pone.0106119.s005.pptx]

## Slide 1
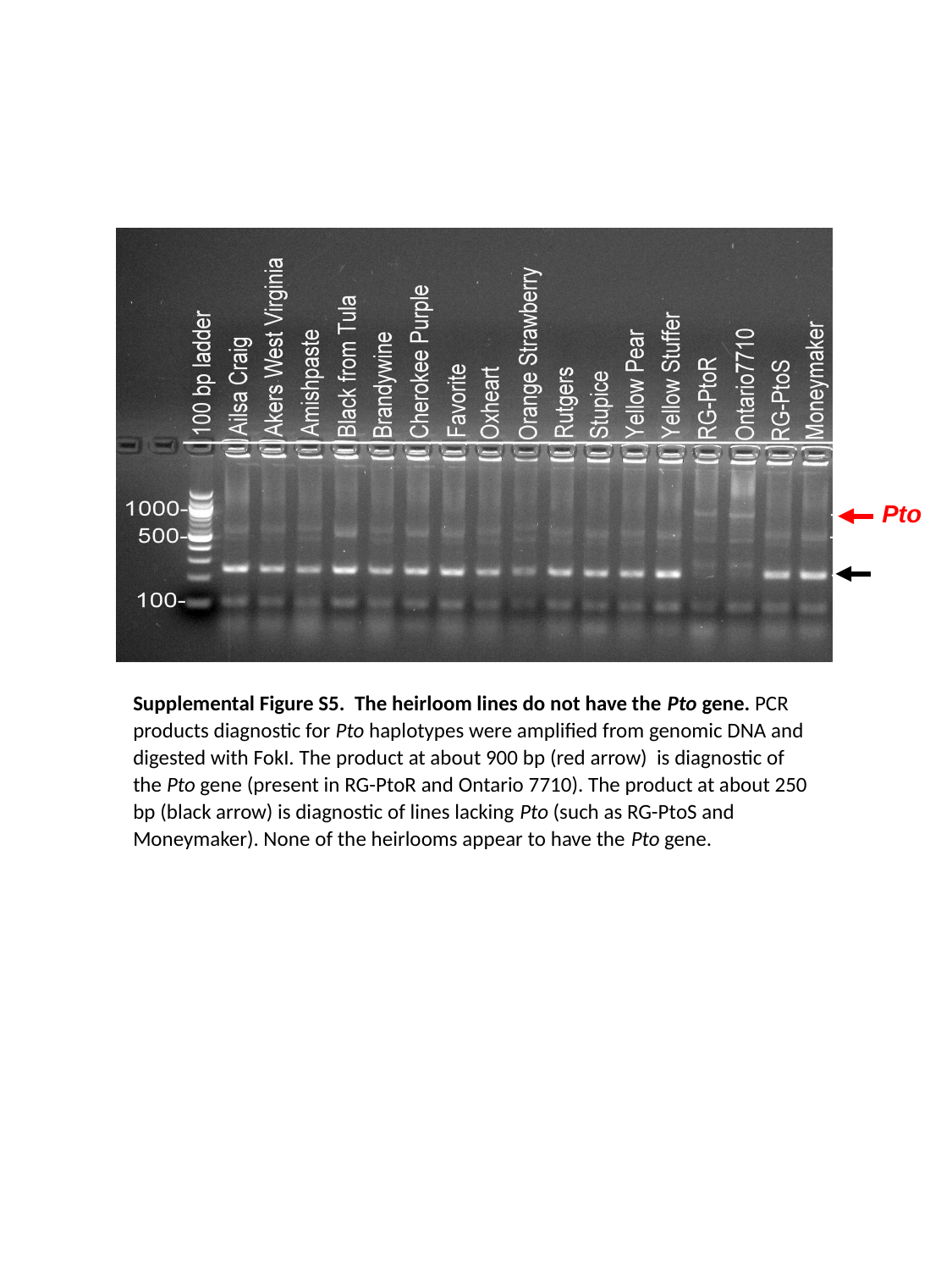

Pto
Supplemental Figure S5. The heirloom lines do not have the Pto gene. PCR products diagnostic for Pto haplotypes were amplified from genomic DNA and digested with FokI. The product at about 900 bp (red arrow) is diagnostic of the Pto gene (present in RG-PtoR and Ontario 7710). The product at about 250 bp (black arrow) is diagnostic of lines lacking Pto (such as RG-PtoS and Moneymaker). None of the heirlooms appear to have the Pto gene.
