# Supplementary material for: Natural Variation for Responsiveness to flg22, flgII-28, and csp22 and Pseudomonas syringae pv. tomato in Heirloom Tomatoes
Source: PLoS One. 2014 Sep 2;9(9):e106119. doi: 10.1371/journal.pone.0106119 (PMC4152135; doi:10.1371/journal.pone.0106119)
Supplement: Figure S6 — Heirloom tomato lines showed differential susceptibility to North Carolina isolate NC-C3. Photos of bacterial speck disease on plants from the field inoculation experiment using North Carolina isolate NC-C3. Photographs were taken on the 7th day after inoculation. Red arrows point to signs of the disease. (PPTX) [file pone.0106119.s006.pptx]

## Slide 1
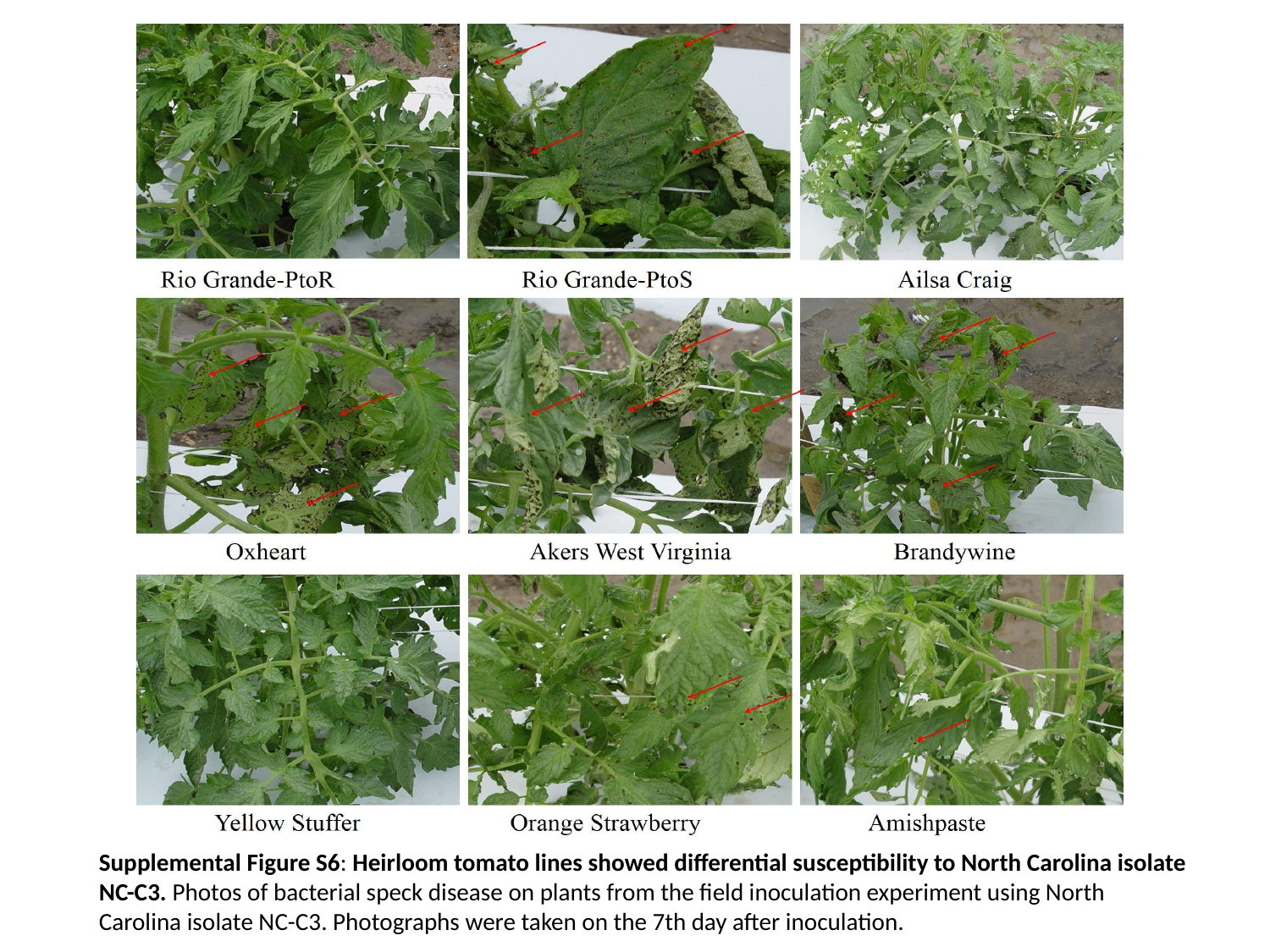

Supplemental Figure S6: Heirloom tomato lines showed differential susceptibility to North Carolina isolate NC-C3. Photos of bacterial speck disease on plants from the field inoculation experiment using North Carolina isolate NC-C3. Photographs were taken on the 7th day after inoculation.
