# Supplementary material for: Natural Variation for Responsiveness to flg22, flgII-28, and csp22 and Pseudomonas syringae pv. tomato in Heirloom Tomatoes
Source: PLoS One. 2014 Sep 2;9(9):e106119. doi: 10.1371/journal.pone.0106119 (PMC4152135; doi:10.1371/journal.pone.0106119)
Supplement: Table S1 — Bacterial isolates from western North Carolina compared with P. s. pv. tomato DC3000. (DOCX) [file pone.0106119.s007.docx]

**Supplemental Table S1:** Bacterial isolates from western North Carolina compared with *P. s.* pv. *tomato* DC3000.

| Isolates | NC county | Fluorescence on KB/UV | RG-PtoR^a^ | RG-PtoS^a^ | Effectors  Present^b^ |
| --- | --- | --- | --- | --- | --- |
| NC-C3 | Henderson | yes | - | +++ | *avrPto, avrPtoB* |
| NC-W201 | Jackson | yes | - | ++ | *avrPto, avrPtoB* |
| DC3000 | - | yes | - | ++ | *avrPto, avrPtoB* |

^a^ Strains were vacuum-infiltrated into the tomato lines RG-PtoR or RG-PtoS and signs of speck disease scored 3 -7 days later. + = disease was observed (severe = +++; less severe = ++) ; - = no disease

^b^ A PCR assay was used to amplify fragments from *avrPto* or *avrPtoB* homologs (see Methods).
