# Supplementary material for: Natural Variation for Responsiveness to flg22, flgII-28, and csp22 and Pseudomonas syringae pv. tomato in Heirloom Tomatoes
Source: PLoS One. 2014 Sep 2;9(9):e106119. doi: 10.1371/journal.pone.0106119 (PMC4152135; doi:10.1371/journal.pone.0106119)
Supplement: Table S2 — DNA primers and PCR conditions used in this study. (DOCX) [file pone.0106119.s008.docx]

**Supplemental Table S2.** Primers and PCR conditions used in this study.

| **Gene** | **Forward primer** | **Reverse primer** | **PCR^a^ (°C)** | **Size** |  |
| --- | --- | --- | --- | --- | --- |
| *avrA* | 5’-CCCTATCCAGCAAGCAGAAG-3’ | 5’-TTGAAGACCAGCGTGCATAG-3’ | 55°C | 1030-bp |  |
| *avrPto* | 5’-ATGGGAAATATATGTGTCGG-3’ | 5’-TCATTGCCAGTTACGGTACGG-3’ | 56^o^C | 492-bp |  |
| *avrPtoB* | 5’-ATGGAACTCTTTCCTGCTC-3’ | 5’-TCAGGGGACTATTCTAAAAGC-3’ | 56^o^C | 1659-bp |  |
| *fliC* | 5’-ATGGCTTTAACAGTAAACACCAAC-3’ | 5’-TTACTGAAGCAGTTTCAGTACAGC-3’ | 55°C | 849-bp |  |
| *hopW1* | 5’ATGAATCCAGCTCAGATCAGGT-3’ | 5’-GAATGACGTAACCCCGTGTATT-3’ | 55°C | 1480-bp |  |
| *cfa7* | 5′-GGACTCAGCAGTATCATCTCGGGACG-3′ | 5′-TGCAGGGTCTTGGGGAGCACG-3′ | 61°C | 689-bp |  |

**^a^**Standard PCR reactions included a 4-min denaturation step at 94°C; followed by 35 cycles of denaturation (94°C), annealing mentioned for each primer set and extension (72°C) and each for 1 min.
