# Supplementary material for: Natural Variation for Responsiveness to flg22, flgII-28, and csp22 and Pseudomonas syringae pv. tomato in Heirloom Tomatoes
Source: PLoS One. 2014 Sep 2;9(9):e106119. doi: 10.1371/journal.pone.0106119 (PMC4152135; doi:10.1371/journal.pone.0106119)
Supplement: Table S3 — Comparison of features of the North Carolina isolates with other well-characterized P. s. pv. tomato strains. (DOCX) [file pone.0106119.s009.docx]

**Supplemental Table S3.** Comparison of features of the North Carolina isolates with other well-characterized *P. s.* pv. *tomato* strains.
